# Early Loss of Fat Mass During Chemoradiotherapy Predicts Overall Survival in Locally Advanced Squamous Cell Carcinoma of the Lung, but Not in Locally Advanced Squamous Cell Carcinoma of the Head and Neck

**DOI:** 10.3389/fnut.2020.600612

**Published:** 2020-11-26

**Authors:** A. C. H. Willemsen, J. H. R. J. Degens, L. W. J. Baijens, A-M. C. Dingemans, A. Hoeben, F. J. P. Hoebers, D. K. M. De Ruysscher, A. M. W. J. Schols

**Affiliations:** ^1^Division of Medical Oncology, Department of Internal Medicine, Maastricht University Medical Center+, Maastricht, Netherlands; ^2^GROW School of Oncology and Developmental Biology, Maastricht University Medical Center+, Maastricht, Netherlands; ^3^Department of Respiratory Medicine, Maastricht University Medical Center+, Maastricht, Netherlands; ^4^NUTRIM School of Nutrition and Translational Research in Metabolism, Maastricht University Medical Center+, Maastricht, Netherlands; ^5^Department of Otorhinolaryngology, Head and Neck Surgery, Maastricht University Medical Center+, Maastricht, Netherlands; ^6^Department of Radiation Oncology (MAASTRO), Maastricht University Medical Center+, Maastricht, Netherlands

**Keywords:** hand grip strength (HGS), locally advanced (stage III) non-small cell lung cancer, locally advanced head and neck cancer, bioelectrical impedance analysis, cachexia, weight loss, chemoradiotherapy (CRT)

## Abstract

**Background:** Cancer cachexia is highly prevalent in advanced non-small cell lung cancer (NSCLC) and locally advanced head and neck squamous cell carcinoma (LAHNSCC), and compromises treatment tolerance and overall survival (OS). NSCLC and LAHNSCC patients share similar risk factors, and receive comparable anti-cancer treatment regimens. The aim of this study was to determine the predictive value of body composition assessed by bioelectrical impedance analysis (BIA) and handgrip strength (HGS) (baseline and early changes during therapy) on OS in NSCLC and LAHNSCC patients treated with platinum-based chemoradiotherapy (CRT) or cetuximab-based bioradiotherapy (BRT). To elucidate potential underlying determinants of early changes in body composition and HGS, specific (fat and fat free) mass loss patterns of squamous NSCLC (sNSCLC) were compared to human papilloma virus negative (HPV–) LAHNSCC patients treated with CRT.

**Methods:** Between 2013 and 2016, BIA and HGS were performed at baseline and after 3 weeks of CRT/BRT in LAHNSCC and NSCLC patients treated with curative intent.

**Results:** Two hundred thirty-three patients were included for baseline measurements. Fat free mass index (FFMI) and HGS<10th percentile of reference values at baseline were both prognostic for poor OS in NSCLC and LAHNSCC [HR 1.64 [95%CI 1.13–2.39], *p* = 0.01 and HR 2.30 [95%CI 1.33–3.97], *p* = 0.003, respectively], independent of Charlson Comorbidity Index, cancer site, and gross tumor volume. Early fat mass (FM) loss during CRT was predictive for poor OS in sNSCLC (*n* = 64) [HR 3.80 [95%CI 1.79–8.06] *p* ≤ 0.001] but not in HPV– LAHNSCC (*n* = 61). In patients with significant weight loss (>2%) in the first 3 weeks of CRT (sNSCLC *n* = 24, HPV– LAHNSCC *n* = 23), the FM change was −1.4 ± 14.5% and −8.7 ± 9.0% in sNSCLC and HPV– LAHNSCC patients, respectively (*p* < 0.05). Fat fee mass change was −5.6 ± 6.3% and −4.0 ± 4.3% for sNSCLC and HPV– LAHNSCC, respectively (*p* = 0.31).

**Conclusion:** FFMI and HGS<10th percentile at baseline are independent prognostic factors for poor OS in NSCLC and LAHNSCC patients treated with CRT/BRT. The specific composition of mass loss during first 3 weeks of CRT significantly differs between sNSCLC and HPV– LAHNSCC patients. Early FM loss was prognostic in sNSCLC only.

## Introduction

In clinical practice, patients with head and neck squamous cell carcinoma (HNSCC) and non-small cell lung cancer (NSCLC) share comparable disease and patient characteristics. For example, smoking is a risk factor for developing both cancer types and histopathologically, in case of squamous cell carcinoma (SCC), one cannot easily distinguish HNSCC from NSCLC (1, 2). Furthermore, the prevalence of comorbidity such as chronic obstructive pulmonary disease (COPD) and cardiovascular disease is high in both HNSCC and NSCLC (3–6). Before the introduction of immunotherapy, standard treatment of locally advanced NSCLC (stage III) included platinum-based chemoradiotherapy (CRT) with disappointing 3 and 5-year overall survival (OS) rates of 43 and 30% (7). However, addition of treatment with durvalumab after concurrent CRT has improved OS to a 3-year OS rate of 57% (8). CRT regimens are also being used in patients with locally advanced HNSCC (LAHNSCC, stage III-IV), and their 5-year OS rates of 34–49% are slightly higher (9, 10). To improve success rates of CRT, it is highly desirable that patients' physical condition and body composition is optimal upon start and maintained during treatment, so patients are more likely to complete the planned treatment trajectory without interruptions of chemotherapy and/or radiation dose reduction due to grade 3–4 toxicity (11–15).

The efficacy of treatment is not only dependent on tumor aspects, but research in many cancer populations has shown that individual patient characteristics including body weight and body composition play an important role. Cancer cachexia, a multifactorial syndrome characterized by an ongoing loss of skeletal muscle mass, (16) is known for being a negative prognostic factor in a wide range of cancer patients, including locally advanced NSCLC and LAHNSCC (17). A significant adverse effect of early weight loss during CRT on OS was observed in stage III NSCLC patients, independent of the onset of therapy induced esophagitis (18–21). In LAHNSCC, significant weight loss was also observed in the first 3 weeks of CRT, which is before onset of the expected therapy induced oral and pharyngeal mucositis (22, 23). In addition to body weight, data on body composition and the presence of sarcopenia/cancer cachexia, could provide information on the expected prognosis of the patient and may therefore support the multidisciplinary team in clinical decision-making. Loss of skeletal muscle mass on CT scans is a strong predictor for poor prognosis in both NSCLC and head and neck cancer (24–26). However, this technique is expensive and time consuming and therefore not routinely feasible in daily clinical practice. Bioelectrical impedance analysis (BIA) combined with handgrip strength (HGS) measurements can easily be implemented in daily clinical care and in a community setting to assess fat mass (FM), fat free mass (FFM), and muscle strength, during treatment (27). Studies have shown that loss of FM and FFM during therapy measured by BIA were correlated to loss of health-related quality of life (28, 29). However, the effect of specific (fat or fat free) body mass loss during the first 3 weeks of treatment on OS, measured by BIA and HGS, has not been investigated for locally advanced NSCLC and LAHNSCC. The aim of this study was to assess the relationship between FM, FFM, and HGS at baseline and changes hereof during first 3 weeks of treatment on the one hand vs. OS on the other hand in advanced cancer patients treated with concurrent CRT or cetuximab-based bioradiation (BRT) with curative intent. In order to clarify potential underlying determinants of changes in body composition and HGS, we also compared specific (fat and fat free) mass loss patterns of NSCLC to LAHNSCC patients. Because differences in histology (squamous vs. non-squamous), tumorgenesis (human papilloma virus positive (HPV+) vs. HPV negative (HPV–), and treatment (cetuximab vs. platinum based) may influence metabolism and therapy response (30–33), this analysis is performed in a more homogeneous subgroup of squamous NSCLC (sNSCLC) and HPV– LAHNSCC patients treated with platinum-based CRT. We hypothesize that a low fat free mass index (FFMI), as a proxy for total body muscle mass, before start of treatment is a negative predictor for OS in both locally advanced NSCLC and LAHNSCC. We expect total weight loss and in particular FM loss to be higher in LAHNSCC due to an additional impaired oral intake in a subset of these patients, depending on tumor site, the presence of oropharyngeal dysphagia, prior head and neck surgery, and dental extraction prior to receiving high dose radiotherapy (34, 35). Presuming comparable treatment induced systemic catabolic activity in LAHNSCC and NSCLC patients, we expect no differences between the groups in FFM changes during the first 3 weeks of CRT.

## Materials and Methods

### Study Design and Population

Patients were followed as part of a larger prospective cohort study conducted at MAASTRO Clinic, Maastricht University Medical Center (MUMC+), Maastricht, the Netherlands (ClinicalTrials.gov Identifier: NCT01985984, LAHNSCC population only). The Institutional Review Board of MAASTRO Clinic approved the study. Patients with stage III NSCLC were referred from four different hospitals in the Netherlands to MAASTRO Clinic for radiotherapy as part of a concurrent platinum-based CRT protocol with curative intent between January 2013 and June 2015. Patients with LAHNSCC undergoing primary or adjuvant concurrent CRT/BRT with curative intent in MAASTRO Clinic and MUMC+ between 2013 and 2016 were included. All patients with body weight assessment, BIA measurements, and HGS measurements at baseline and during week 3 of treatment were included in the study. Patients presenting concurrent malignancies beside the locally advanced NSCLC or LAHNSCC were excluded.

First, the effect of baseline characteristics on OS (part I) was evaluated in NSCLC and LAHNSCC patients undergoing radiotherapy and any concurrent systemic therapy.

In order to elucidate possible underlying mechanisms contributing to weight loss in the first 3 weeks of therapy in both patient groups (part II), exclusion of potential confounders was considered essential. Only patients with SCC were included in the second part of this study. LAHNSCC patients receiving cetuximab as radiosensitizer, and tumors that were positive for P16 (P16+), a surrogate marker for HPV, were excluded. In this cohort, the P16 status was available for all oropharyngeal tumors, but subsequent HPV RT-PCR analysis was not performed in all cases. For the convenience of the reader, P16+ tumors are referred to as HPV+ in this manuscript.

### Oncological Treatment

For NSCLC, the concurrent systemic therapy consisted of three cycles of cisplatin (100 mg/m^2^ q3w) or carboplatin (AUC5 on day 1) in combination with etoposide (100 mg/m2 day 1–3) administered every 3 weeks. Radiotherapy was applied in 30–33 daily fractions of 2 Gy up to a total dose of 60–66 Gy (36, 37).

In LAHNSCC patients, concurrent cisplatin was administered intravenously on days 1, 22, and 43, in doses of 100 mg/m^2^ in case of primary and adjuvant treatment (15, 38). Adjuvant CRT was given in case of extra nodal extension in neck dissections and/or irradical tumor resection margins. In case of contra-indications for cisplatin, and only in a primary treatment setting, cetuximab was administered weekly in doses of 250 mg/m^2^, preceded by a loading dose of 400 mg/m^2^, 1 week before radiotherapy initiation in a primary treatment setting only (14). Radiotherapy was applied in 33–35 fractions up to a total dose of 66–70 Gy.

### Measurements

Body composition was assessed using single-frequency (50 kHz) BIA (Omron Healthcare Group, Hoofddorp, The Netherlands). FFMI was calculated by dividing FFM in kg by height in meters squared. Low FFM was defined as a FFMI below the 10th percentile (FFMI<P_10_), corrected for age and gender according to Schutz et al. (39).

HGS was measured with a Jamar hydraulic hand dynamometer (JA Preston Corporation, Jackson, MI, USA). The measurements were repeated three times for the left and right hand and the highest value for both sides was registered. Follow up HGS was chosen based on the side with the highest value at baseline. Low HGS was defined as HGS below the 10th percentile (HGS<P_10_) of the UK Biobank reference values, taking gender, age and height into account (40).

Weight, BIA and HGS measurements were performed in the outpatient clinic within office hours and prior to chemotherapy infusion to minimalize the influence of chemotherapy and fluid infusion.

World Health Organization Performance Status (WHO PS) was assessed by the radiation oncologist at the initial visit. The Charlson Comorbidity Index (CCI) (41) was determined by review of individual medical records. The current malignant disease was not taken into account when rating “solid tumor” in the CCI. The cut-off for high CCI was set at ≥4.

Weight loss, FM loss, and FFM loss were turned into binary variables, where losses were compared to stable or increased mass. The cut-off for significant weight loss was set on −2% based on recent guidelines (42). To minimize potential effects of measurement errors, the cut-off values for the specific mass losses (FM and FFM) were set at 1% mass loss.

Gross tumor volume (GTV) was retrieved from records of radiation dosimetry data and divided in GTV of primary tumor (GTV_p_), GTV of involved lymph nodes (GTV_n_), and the total GTV of primary tumor and lymph nodes combined (GTV_total_). For LAHNSCC patients, all GTV data were retrieved from patients that underwent primary CRT/BRT. In patients undergoing post-operative CRT, GTV's were only registered in case of tumor residue or regrowth in the period between surgery and start of radiotherapy.

### Statistical Analysis

Statistical analyses were performed using IBM SPSS Statistics for Windows, Version 25 (IBM Corp., Armonk, New York, USA). Descriptive statistics were reported in frequency distributions and absolute numbers by using independent samples *t-*test and χ^2^ test. Paired samples *t-*test was used to evaluate mass loss.

Survival analysis was performed through Kaplan Meier (Logrank Mantel–Cox test) and Cox regression. All variables were screened for their effect on OS through univariable cox regression. Factors with *p* < 0.30 were selected as potentially relevant predictor variables and were entered in a multivariable cox regression model. Stepwise backward elimination was carried out to omit all variables without an effect on OS from the model using a *p*-value for selection of 0.10.

## Results

### Patients and Baseline Characteristics

Between January 2013 and July 2015, 172 patients with pathologically proven stage III NSCLC were referred to MAASTRO Clinic for concurrent CRT. In 124 patients, HGS and BIA measurements were performed at baseline and during treatment (part I). Sixty-four of the 124 NSCLC patients had histologically proven SCC and were included in part II of the study. Hundred-ninety-two LAHNSCC patients were treated with primary or adjuvant CRT/BRT between 2013 and 2016. Baseline and follow-up measurements in week 3 of CRT were available in 109 patients (part I), of which 61 were HPV– tumors receiving cisplatin as radiosensitizer (part II). Table 1 summarizes the baseline patient characteristics.

**Table 1:** Baseline characteristics (*n* = 233).

**Variables**		
**Gender**	Male	159
	Female	74
**Age**	Mean ± SD, years	62 ± 9
**Smoking status**	Active/former	214
	Never	14
	Unknown	5
**Cancer site**	Lung	124
	Head and neck	109
**Histology**	Squamous	173
	Non-squamous	60
**GTV**_**P**_	Total (Mean ± SD, cm^3^)	52.1 ± 88.4
	Squamous NSCLC	86.2 ± 131.9
	Non-squamous NSCLC	69.4 ± 86.5
	Primary LAHNSCC	29.3 ± 35.5
	Post-op LAHNSCC*	4.6 ± 4.4
**GTV**_**N**_	Total (Mean ± SD, cm^3^)	19.0 ± 26.5
	Squamous NSCLC	24.9 ± 30.6
	Non-squamous NSCLC	23.4 ± 23.3
	Primary LAHNSCC	16.5 ± 27.0
	Post-op LAHNSCC*	8.9 ± 14.8
**GTV**_**TOTAL**_	Total (Mean ± SD, cm^3^)	71.3 ± 91.8
	Squamous NSCLC	111.0 ± 132.9
	Non-squamous NSCLC	93.4 ± 84.6
	Primary LAHNSCC	45.8 ± 41.7
	Post-op LAHNSCC*	13.5 ± 14.1
**Mean heart dose (NSCLC only)**	Total (Mean ± SD, Gy)	9.0 ± 8.0
	Squamous NSCLC	9.1 ± 8.5
	Non-squamous NSCLC	8.9 ± 7.4
**Systemic therapy**	Platinum + etoposide (NSCLC)	124
	Platinum (LAHNSCC)	82
	Cetuximab (LAHNSCC)	27
**BMI** (kg/m^2^)	Total	24.4 ± 4.2
	Male	24.8 ± 4.1
	Female	23.5 ± 4.4
**FMI** (kg/m2)	Total	6.8 ± 2.8
	Male	6.4 ± 2.6
	Female	7.7 ± 3.2
**FFMI** (kg/m^2^)	Total	17.6 ± 2.5
	Male	18.4 ± 2.2
	Female	15.8 ± 1.9
**FFMI**	< P_10_	71
	P_10_ or higher	162
**HGS**	< P_10_	20
	P_10_ or higher	213
**WHO PS**	0–1	218
	2	15
**History of COPD**	Yes	165
	No	66
	Unknown	2
**CCI**	0–3	175
	4 or higher	58
**Disease stage** *NSCLC only*	IIIA	52
	IIIB	53
	IIIC	19
**Disease stage** *LAHNSCC only*	II	2
	III	19
	IV	88
**Dysphagia** *LAHNSCC only*	CTCAE ≥ 2	30
	CTCAE <2	79
**P16 status** *LAHNSCC only*	Positive oropharynx tumor	23
	Others	86
**Adjuvant or primary** *LAHNSCC only*	Primary	86
	Adjuvant	23

Disease stage is based on TNM 7 classification (43). GTV_p_, gross tumor volume of primary tumor; GTV_n_, gross tumor volume of lymph nodes involved; GTV_total_, combined GTV of both primary tumor and lymph nodes; BMI, body mass index; FMI, fat mass index; FFMI, fat free mass index; P_10_, tenth percentile according to reference values Spruit (40) and Schutz (39); HGS, handgrip strength; WHO PS, world health organization performance status; CCI, Charlson comorbidity index; COPD, chronic obstructive pulmonary disease. P16 is a surrogate marker for Human Papillomavirus infection, Mean values are presented with ± SD. *Only from five patients with tumor residual or regrowth after surgery.

### Part I—Prognostic Value of Baseline Characteristics for Overall Survival

**Figure 1:**
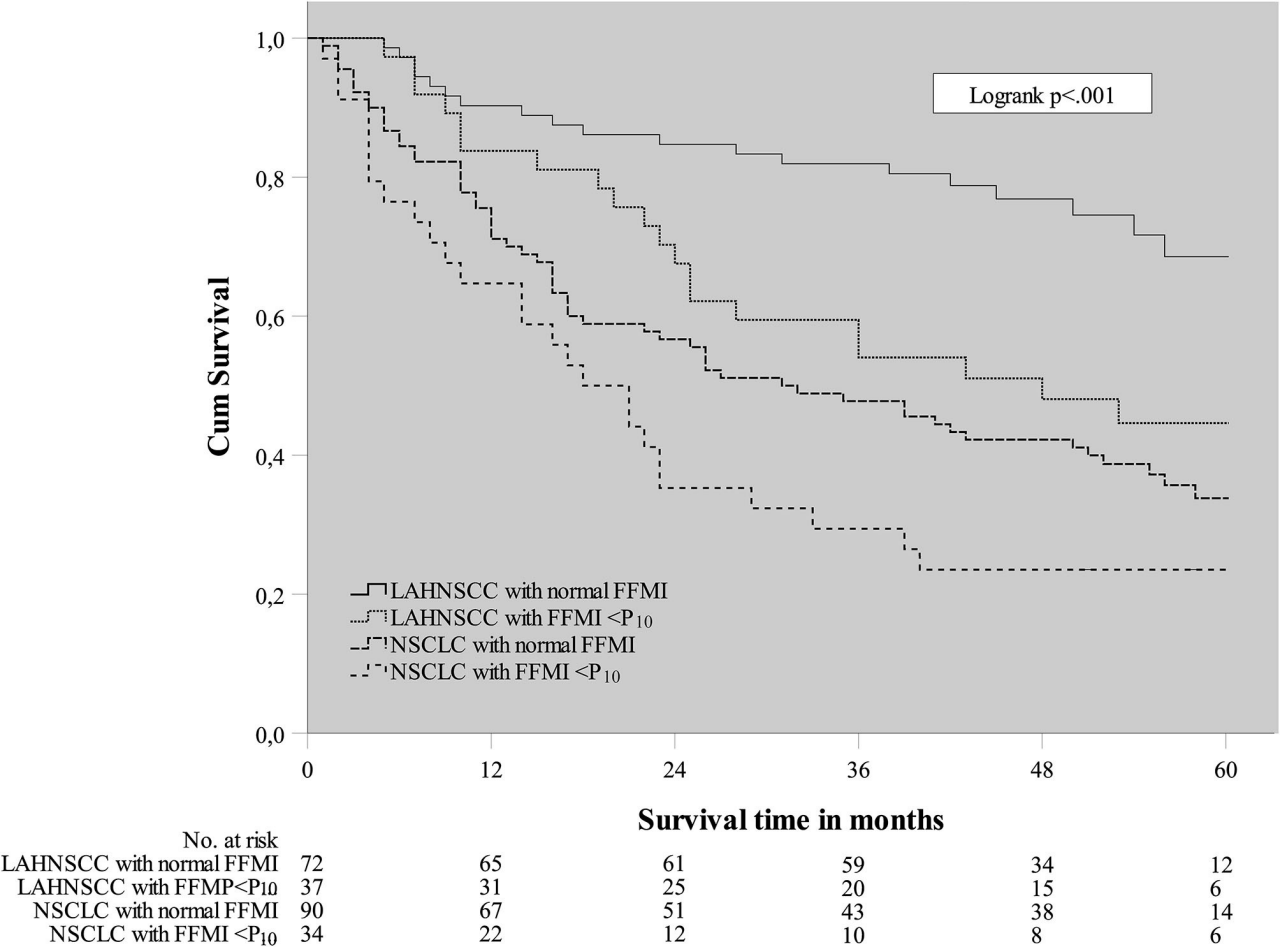
Kaplan Meier survival plot of baseline FFMI<P_10_ per cancer site.

A Cox regression analysis was performed in the total cancer population (*n* = 233) to determine any confounding factors for OS within the baseline characteristics (Table 2). Using univariable analysis for the total population, the following variables yielded a *p* < 0.30 and were considered potentially relevant prognostic factors suitable for multivariate analysis: age, WHO PS, CCI, cancer site (NSCLC vs. LAHNSCC), history of COPD, FFMI<P_10_, HGS<P_10_, and GTV. Using multivariable Cox regression analysis, only CCI≥4, cancer site being NSCLC, FFMI<P_10_, HGS<P_10_, and GTV_total_ remained statistically significant and were considered independent prognostic factors for OS. Kaplan Meier survival curve plotted in Figure 1 shows significant differences in OS for four different categories: LAHNSCC patients with normal FFMI, LAHNSCC patients with FFMI<P_10_, NSCLC patients with normal FFMI, and NSCLC patients with FFMI<P_10_ (Logrank Mantel Cox *p* < 0.001). Two and 5 year OS rate for LAHNSCC patients with normal FFMI was 85 and 69%, respectively, compared to 68 and 45% for LAHNSCC patients with FFMI<P_10_. For NSCLC patients, 2 and 5 year OS rate was 57 and 34%, respectively, for those with normal FFMI, compared to 35 and 24% for NSCLC patients with FFMI<P_10_.

**Table 2:** Cox regression analysis of baseline characteristics on OS (*n* = 233).

	**Univariate analysis**				**Multivariate analysis**			
	**95% CI**				**95% CI**			
**Covariate**	**HR**	**Lower**	**Upper**	***p-*value**	**HR**	**Lower**	**Upper**	***p-*value**
Male gender	1.10	0.75	1.62	0.62				
Age	1.03	1.01	1.06	**0.004**				
WHO PS (≥ 2)	2.49	1.37	4.53	**0.003**	1.78	0.94	3.38	0.08
CCI ≥ 4	1.89	1.30	2.76	**0.001**	1.52	1.02	2.27	**0.04**
Active/past smoker	1.13	0.60	2.12	0.70				
Cancer site (NSCLC)	2.51	1.72	3.67	** <0.001**	2.12	1.40	3.22	** <0.001**
History of COPD*	1.35	0.93	1.97	0.12				
FFMI ≤ P10	1.61	1.12	2.31	**0.01**	1.64	1.13	2.39	**0.01**
FMI ≤ P10	1.06	0.63	1.79	0.83				
HGS<p10	2.51	1.48	4.26	**0.001**	2.30	1.33	3.97	**0.003**
GTV_p_	1.003	1.001	1.004	**0.001**				
GTV_n_	1.009	1.004	1.014	**0.001**				
GTV_total_	1.003	1.002	1.005	** <0.001**	1.002	1.000	1.004	**0.02**

Disease stage is based on TNM 7 classification (43). *Two missing. WHO PS, world health organization performance status; CCI, Charlson comorbidity index; COPD, chronic obstructive pulmonary disease; FMI, fat mass index; FFMI, fat free mass index; P_10_, tenth percentile according to reference values Spruit (40) and Schutz (39); HGS, handgrip strength; GTV_p_, gross tumor volume of primary tumor; GTV_n_, gross tumor volume of lymph nodes involved; GTV_total_, combined GTV of both primary tumor and lymph nodes. Underlined values indicate p value below 0.30. Bold values indicate statistical significance at the p value of 0.050.

### Part II—Fat Mass, Fat Free Mass, and Handgrip Strength Changes

As stated in the method section, a subanalysis was performed to rule out potential confounders affecting weight loss. Supplementary Table 1 provides an overview of baseline characteristics of the 64 sNSCLC and 61 HPV– LAHNSCC patients. No statistically significant group differences were observed for the variables gender, smoking status, BMI, FMI, FFMI, HGS, WHO PS, and history of COPD. Mean GTV's were significantly higher in sNSCLC patients and a CCI ≥ 4 was more prevalent in sNSCLC patients compared to HPV– LAHNSCC patients, which might be explained by an additional significantly younger age of the latter group.

**Figure 2:**
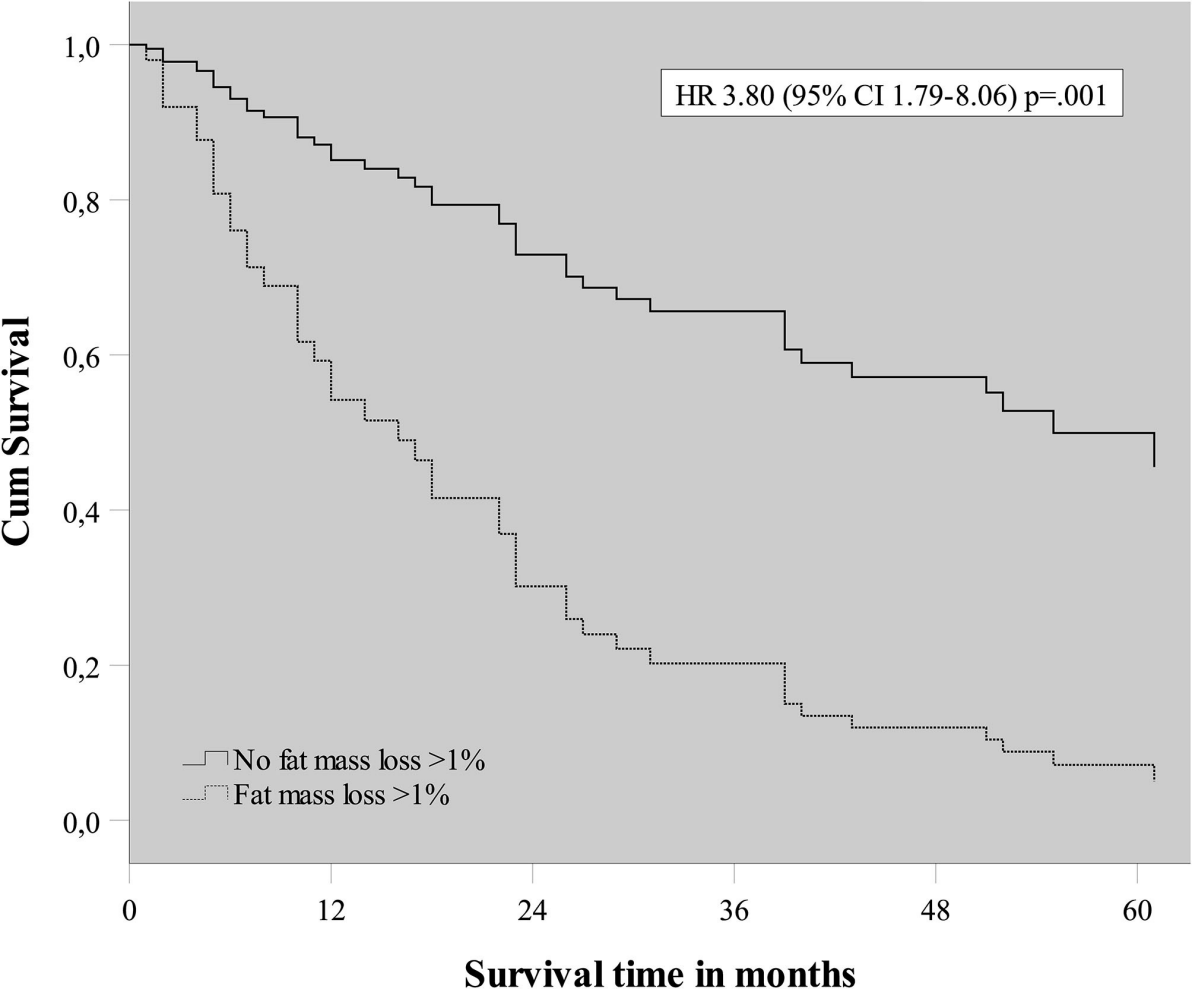
Cox regression survival plot of early fat mass loss in squamous NSCLC.

To evaluate the effect of early mass loss (FM or FFM) on OS, Cox regression analysis was performed for the sNSCLC (*n* = 64) and HPV– LAHNSCC (*n* = 61) subgroup separately. Multivariable Cox regression analysis in the sNSCLC population showed the independent prognostic value of age [HR 1.08 [95%CI 1.03–1.14] *p* = 0.001], WHO PS ≥ 2 [HR 2.59 [95%CI 1.05–6.36]] *p* = 0.04], FM loss >1% in first 3 weeks of therapy [HR 3.80 [95%CI 1.79–8.06] *p* = 0.001], and GTV_n_ [HR 1.016 [95%CI 1.006–1.026] *p* = 0.002]. A trend of worse OS could be observed in case of FFM loss >1% [HR 1.85 [95%CI 0.90–3.82] *p* = 0.09] (Table 3A and Figure 2). In HPV– LAHNSCC patients treated with CRT, multivariable analysis displayed no independent prognostic factors (Table 3B).

**Table 3A:** Cox regression analysis of early mass loss in sNSCLC (*n* = 64).

	**Univariate analysis**				**Multivariate analysis**			
	**95% CI**				**95% CI**			
**Covariate**	**HR**	**Lower**	**Upper**	***p-*value**	**HR**	**Lower**	**Upper**	***p-*value**
Male gender	0.99	0.47	2.05	0.97				
Age	1.06	1.01	1.11	0.01	1.08	1.03	1.14	**0.001**
WHO PS (≥ 2)	3.03	1.33	6.88	0.008	2.59	1.05	6.36	0.04
CCI ≥ 4	1.35	0.74	2.48	0.33				
Active/past smoker	1.25	0.37	4.18	0.72				
History of COPD	1.25	0.68	2.30	0.47				
Disease stage								
IIIA				0.53				
IIIB	1.34	0.67	2.66	0.41				
IIIC	1.53	0.70	3.34	0.29				
FFM loss >1%	1.73	0.94	3.15	0.08	1.85	0.90	3.82	**0.09**
FM loss >1%	2.23	1.23	4.06	0.008	3.80	1.79	8.06	**0.001**
HGS loss >1%	0.87	0.48	1.57	0.64				
GTVp	1.001	0.998	1.004	0.46				
GTVn	1.008	1.001	1.016	**0.03**	1.016	1.006	1.026	**0.002**
GTVpn	1.002	0.999	1.004	0.18				
Mean heart dose	1.029	0.995	1.066	0.10				

*Disease stage is based on TNM 7 classification; Underlined values indicate p value below 0.30. Bold values indicate statistical significance at the p value of 0.050. WHO PS, world health organization performance status; CCI, Charlson comorbidity index; COPD, chronic obstructive pulmonary disease; FFM, fat free mass; FM, fat mass; HGS, handgrip strength; GTV_p_, gross tumor volume of primary tumor; GTV_n_, gross tumor volume of lymph nodes involved; GTV_total_, combined GTV of both primary tumor and lymph nodes*.

**Table 3B:** Cox regression analysis of early mass loss in HPV– LAHNSCC receiving platinum based systemic therapy (*n* = 61).

	**Univariate analysis**				**Multivariate analysis**			
	**95% CI**				**95% CI**			
**Covariate**	**HR**	**Lower**	**Upper**	***p-*value**	**HR**	**Lower**	**Upper**	***p-*value**
Male gender	2.93	0.87	9.45	0.08	2.93	0.87	9.45	0.08
Age	0.98	0.93	1.02	0.34				
WHO PS (≥ 2)	1.53	0.21	11.5	0.68				
CCI ≥ 4	1.34	0.18	10.0	0.77				
Active/past smoker	0.48	0.14	1.62	0.24				
History of COPD	1.09	0.42	2.78	0.86				
Disease stage								
II				0.80				
III	0.39	0.04	3.50	0.40				
IVa	0.57	0.08	4.34	0.59				
IVb	0.37	0.02	5.97	0.48				
Dysphagia CTCAE ≥ 2	0.87	0.32	2.35	0.78				
Adjuvant	0.99	0.40	2.4	0.98				
FFM loss >1%	1.03	0.45	2.39	0.94				
FM loss >1%	1.63	0.60	4.41	0.34				
HGS loss >1%	1.25	0.51	3.06	0.63				
GTV_p_	1.004	0.996	1.012	0.33				
GTV_n_	1.005	0.980	1.031	0.68				
GTV_total_	1.004	0.996	1.012	0.28				

*Disease stage is based on TNM 7 classification; Underlined values indicate p value below 0.30. WHO PS, world health organization performance status; CCI, Charlson comorbidity index; COPD, chronic obstructive pulmonary disease; CTCAE, common terminology criteria of adverse events; FFM, fat free mass; FM, fat mass; HGS, handgrip strength; GTV_p_, gross tumor volume of primary tumor; GTV_n_, gross tumor volume of lymph nodes involved; GTV_total_, combined GTV of both primary tumor and lymph nodes*.

The proportion of patients experiencing significant weight loss (>2%) during first 3 weeks of therapy did not significantly differ between sNSCLC and HPV– LAHNSCC, 24/64 vs. 23/61, respectively (*p* = 0.98). In these weight losing patients, proportional weight loss did not significantly differ between sNSCLC and LAHNSCC, −4.8 ± 2.6% (mean ± SD) and −5.2 ± 2.1%, respectively *p* = 0.62. However, when distinguishing the composition of body mass loss, a significant difference was observed: FM change was −1.4 ± 14.5% and −8.7 ± 9.0% in sNSCLC and HPV– LAHNSCC patients, respectively (*p* < 0.05). FFM change was −5.6 ± 6.3 and −4.0 ± 4.3% for sNSCLC and HPV– LAHNSCC, respectively (*p* = 0.31). Specification of the composition of body mass loss for the total 125 patients is provided in the Supplementary Table 2.

## Discussion

This study was conducted to elucidate the effect of body composition and HGS before start of treatment and early changes of FM, FFM, and HGS during CRT on OS in patients diagnosed with NSCLC or LAHNSCC.

HGS and FFMI <P_10_ at baseline as a surrogate marker for muscle wasting were both negative prognostic factors for OS, independent of cancer site (NSCLC or LAHNSCC) and GTV. This is in full accordance with the current literature on muscle wasting in cancer patients (44–46). While the effect of muscle wasting on OS is not new, our results emphasize that simple measurements such as BIA and HGS can detect muscle wasting in NSCLC and LAHNSCC patients in an early phase of treatment.

Previously, our group revealed the prognostic significance of early weight loss in NSCLC patients (20). In the current study, we confirmed this finding and unraveled the components of this early weight loss and the effect on OS. Strikingly, in multivariable Cox regression analysis, FM loss remained a significant prognostic factor for OS in sNSCLC, but not in HPV– LAHNSCC patients.

The analysis of specific mass loss patterns was carried out presuming comparable patient characteristics between both cancer populations, by leaving out adenocarcinoma, HPV+ LAHNSCC, and cetuximab as systemic therapy (part II). However, our analysis revealed a higher CCI in sNSCLC, as a result of an additional higher mean age of this group, which is a parameter in the CCI calculation. Also, GTV was higher in NSCLC compared to LAHNSCC. Yet, despite these differences, body composition parameters were similar for both groups, which allowed further comparison.

While we hypothesized to find a higher weight loss in LAHNSCC due to tumor location related factors impairing oral intake, our results show otherwise. The percentage of early weight loss (first 3 weeks of CRT) was comparable between NSCLC and LAHNSCC patients, and the proportion of patients that experienced significant weight loss (>2%) was similar between both cancer sites.

Nevertheless, evaluating the composition of weight loss, in particular FM and FFM loss, our data revealed different patterns for both sub groups. CRT receiving HPV– LAHNSCC patients with significant weight loss seemed to lose FM in particular, which was significantly higher than in sNSCLC patients. sNSCLC patients with significant weight loss (>2%) seem to lose predominantly FFM. Both sNSCLC and HPV– LAHNSCC patients experiencing significant weight loss showed a decrease in FFM, with a trend toward higher FFM loss in sNSCLC patients.

We believe potential explanations for the differences in body composition changes and the prognostic value of early weight loss are multifactorial, including patient characteristics, tumor metabolism and load, and treatment related factors.

The higher CCI and the higher prevalence of COPD in sNSCLC patients suggest an additional burden on systemic inflammation, which is known to contribute to muscle wasting (47). However, one would also expect baseline group differences in body composition, which were not observed. Additionally, there could be an underestimation of the COPD prevalence in LAHNSCC patients as these patients are not routinely tested for COPD. Furthermore, when evaluating the subgroup of HPV– LAHNSCC patients receiving cisplatin as radiosensitizer (part II), the CCI may be lower than in sNSCLC due to the strictly applied selection criteria for receiving platinum-based CRT in LAHNSCC: In case of significant (cardiac, pulmonary, and renal) comorbidity, platinum based treatment is withheld in LAHNSCC and cetuximab is used as radiosensitizer. The latter was excluded in the subanalysis of part II. Therefore, organ dysfunction related to comorbidities is probably barely present in the HPV– LAHNSCC treated with cisplatinum-based CRT subgroup, in contrast to the sNSCLC patients, where carboplatin is offered in case of contraindications for cisplatin (48).

Tumor characteristics such as location, size, and tumor metabolism differ between the sNSCLC and HPV– LAHNSCC group. The higher amount of FM loss in LAHNSCC might be due to oral intake-related factors (tumor location, prior head and neck surgery, odynophagia, or dysphagia) rather than high catabolic activity causing muscle wasting, which was more pronounced in NSCLC. Early dietary consultation in LAHNSCC has a positive effect on weight maintenance (49). Conceivably, administration of oral nutritional supplements or tube feeding in LAHNSCC may have blurred the effects of weight loss on OS. Unfortunately, reliable information on the exact nutritional support given could not be retrieved from the medical records. Interventions to improve or maintain body weight and muscle mass in particular are under investigation, but have not resulted in standardized supportive treatment protocols yet. The current study results support the need for further research into interventions for these patient groups.

Yet, a key point in cancer cachexia is that it cannot be fully reversed by nutritional support, and systemic inflammation plays an important role in both muscle and FM loss (50). Both tumor and host tissue can produce cytokines leading to inflammation. It has been reported that systemic inflammation is correlated with tumor volume (51) and previous studies have shown the prognostic value of GTV (52). Our results show the prognostic value of GTV independent of baseline FFMI<P_10_, suggesting higher systemic inflammation in more voluminous tumors (51).

It is likely that also oncological treatment related factors play a role in the prognostic value of early mass loss in NSCLC but not in LAHNSCC. While concurrent systemic therapy for LAHNSCC consists of cisplatin only, platinum derivatives combined with etoposide are used for NSCLC. Platinum-based regimens are known to induce the release of pro-cachectic cytokines and myostatin, activating the nuclear factor kappa-light-chain-enhancer of activated B cells (NF-κB) signaling pathway, which is associated with muscle wasting (53). Etoposide has also shown to induce muscle wasting (54). The combination of both drugs might accelerate muscle wasting in NSCLC. Also, GTV was higher in NSCLC vs. LAHSNCC, meaning that more cells will experience DNA-damage during radiation, which can result in a higher pro-inflammatory environment (55).

We are aware that our research has limitations. Generally accepted cut off points for FM and FFM loss have not been defined in the literature. For exploratory purposes, we chose an arbitrary cut-off on an empirical basis. Additionally, standardized information on oral intake and the use of nutritional supplements or tube feeding was lacking. Evaluating FM through BIA does not provide information on the type of fat being lost, e.g., visceral adipose tissue or subcutaneous adipose tissue. Also, BIA results should be interpreted with caution in case of changes in the patients' hydration status and physical exercise. The authors are aware that due to these limitations, BIA is not considered the gold standard. Furthermore, BIA is not suitable for independently identifying changes in skeletal muscle index. However, the FFMI can be measured using BIA and is a realistic reflection of skeletal muscle mass. The final limitation we would like to address is that our study did not include progression or disease free survival analyses. However, CCI was added in our OS analyses to adjust for the effects of comorbidities on OS.

In conclusion, our study showed that low (<P_10_) HGS and FFMI at baseline are independent prognostic factors for poor OS in NSCLC and LAHNSCC patients treated with CRT/BRT with curative intent. Despite comparable patient characteristics, treatment, and histological tumor characteristics, the specific composition of mass loss during the first 3 weeks of CRT significantly differs between sNSCLC and HPV– LAHNSCC patients and early loss of FM was prognostic in sNSCLC only. Further research is needed to unravel the pathophysiology of early weight loss during oncological treatment in order to develop patient-tailored intervention studies.

## Data Availability Statement

The data analyzed in this study is subject to the following licenses/restrictions: The datasets generated during and/or analyzed during the current study are not publicly available but are available from the corresponding author on reasonable request. Requests to access these datasets should be directed to AW, rianne.willemsen@maastrichtuniversity.nl.

## Ethics Statement

The studies involving human participants were reviewed and approved by Institutional Review Board of MAASTRO Clinic. Written informed consent for participation was not required for this study in accordance with the national legislation and the institutional requirements.

## Author Contributions

AW and JD conducted research, collected data, analyzed data, and wrote the manuscript. AS and A-MD were involved in research design, data analysis, and writing. AH and LB were involved for their expert opinion in data interpretation and translation to clinical practice. FH and DD provided essential material and were involved for their expert opinion in data interpretation and translation to clinical practice. All authors contributed to the article and approved the submitted version.

## Conflict of Interest

The authors declare that the research was conducted in the absence of any commercial or financial relationships that could be construed as a potential conflict of interest.

## Abbreviations

AUC: area under the curve
BIA: bioelectrical impedance analysis
BRT: bioradiotherapy (cetuximab based)
CCI: Charlson comorbidity index
CI: confidence interval
COPD: chronic obstructive pulmonary disease
CRT: chemoradiotherapy (platinum based)
CTCAE: common terminology criteria of adverse events
DNA: deoxyribonucleic acid
FFM: fat free mass
FFMI: fat free mass index
FFMI<P_10_: fat free mass index below the tenth percentile
FM: fat mass
GTV_p_: gross tumor volume of primary tumor
GTV_n_: gross tumor volume of lymph nodes involved
GTV_total_: combined GTV of both primary tumor and lymph nodes
Gy: Gray
HGS: handgrip strength
HNSCC: head and neck squamous cell carcinoma
HPV: human papilloma virus
HR: hazard ratio
LAHNSCC: locally advanced head and neck squamous cell carcinoma
MUMC+: Maastricht University Medical Center +
NF-κB: nuclear factor kappa-light-chain-enhancer of activated B cells
OS: overall survival
SCC: squamous cell carcinoma
SD: standard deviation
sNSCLC: squamous non-small cell lung carcinoma
RT-PCR: reverse transcription polymerase chain reaction
WHO PS: world health organization performance status.
