# Supplementary material for: Early Loss of Fat Mass During Chemoradiotherapy Predicts Overall Survival in Locally Advanced Squamous Cell Carcinoma of the Lung, but Not in Locally Advanced Squamous Cell Carcinoma of the Head and Neck
Source: Front Nutr. 2020 Nov 26;7:600612. doi: 10.3389/fnut.2020.600612 (PMC7726186; doi:10.3389/fnut.2020.600612)
Supplement: Supplementary file 1 [file Data_Sheet_1.docx]

Supplementary Material

**Table S1. Baseline characteristics squamous NSCLC and HPV- LAHNSCC treated with platinum-based systemic therapy.**

| **Variables** | | **LAHNCC**  n=61 (%) | **NSCLC**  n=64 (%) | **P value** |
| --- | --- | --- | --- | --- |
| **Gender** | Male  Female | 43 (70)  18 (30) | 50 (78)  14 (22) | .33 |
| **Mean age** | Mean ± SD, years | 58 ± 8 | 67 ± 8 | **<.001** |
| **Smoking status** | Never  Active/former | 5 (8)  56 (92) | 1 (2)  61 (95)* | .11 |
| **BMI** (kg/m^2^) | Total  Male  Female | 23.9 ± 4.0  23.9 ± 3.8  23.8 ± 4.5 | 24.6 ± 4.0  24.6 ± 4.0  24.7 ± 4.1 | .33  .44  .58 |
| **FMI** (kg/m2) | Total  Male  Female | 6.3 ± 2.6  5.7 ± 2.1  7.8 ± 3.1 | 6.8 ± 2.8  6.3 ± 2.6  8.4 ± 3.0 | .29  .17  .55 |
| **FFMI** (kg/m^2^) | Total  Male  Female | 17.6 ± 2.5  18.3 ± 2.4  16.0 ± 2.2 | 17.8 ± 2.0  18.2 ± 1.9  16.2 ± 1.6 | .65  .94  .78 |
| **FFMI** | <P_10_  P_10_ or higher | 22 (36)  39 (64) | 19 (30)  45 (70) | .57 |
| **HGS** | <P_10_  P_10_ or higher | 3 (5)  58 (95) | 8 (13)  56 (87) | .21 |
| **WHO PS** | 0 - 1  2 | 59 (97)  2 (3) | 57 (89)  7 (11) | .16 |
| **History of COPD** | Yes  No | 16 (26)  45 (74) | 24 (38)  39 (62)** | .16 |
| **CCI** | 0-3  4 or higher | 59 (97)  2 (3) | 40 (63)  24 (37) | **<.001** |
| **Disease stage** | IIIA  IIIB  IIIC | -  -  - | 25 (39)  26 (41)  13 (20) | - |
| **Disease stage** | II  III  Iva  IVb | 2 (3)  14 (23)  42 (69)  3 (5) | -  -  - | - |
| **GTV_p_** | Mean ± SD, cm^3^ | 31.9 ± 41.4 | 86.2 ± 131.9 | **.01** |
| **GTV_n_** | Mean ± SD, cm^3^ | 11.4 ± 18.7 | 24.9 ± 30.6 | **.01** |
| **GTV_total_** | Mean ± SD, cm^3^ | 43.3 ± 42.2 | 111.0 ± 132.9 | **.002** |
| **Mean heart dose** | Mean ± SD, Gy | - | 9.1 ± 5.7 | **-** |
| **Dysphagia** | CTCAE ≥ 2  CTCAE <2 | 15 (25)  46 (74) | -  - | - |
| **Adjuvant or primary** | Primary  Adjuvant | 42 (69)  19 (31) | -  - | - |

Percentages do not always add up to 100% due to rounding off to whole numbers. Continuous numbers are presented with ± SD.

*two missing, **One missing

Disease stage is based on TNM 7 classification. BMI, body mass index; FMI, fat mass index; FFMI, fat free mass index; P_10_, tenth percentile according to reference values Spruit and Schutz; HGS, handgrip strength; WHO PS, world health organization performance status; CCI, Charlson comorbidity index; COPD, chronic obstructive pulmonary disease, GTV_p_, gross tumor volume of primary tumor; GTV_n_, gross tumor volume of lymph nodes involved, GTV_total_ combined GTV of both primary tumor and lymph nodes. For LAHNSCC, GTV is only displayed for the 42 patients treated with primary CRT.

**Table S2. Percentage changes in weight, fat mass, fat free mass and handgrip strength in squamous NSCLC and HPV- LAHNSCC, treated with platinum based systemic therapy.**

| **Variables** | **Total population**  (n=125) | ***p* value** | **LAHNSCC**  (n=61) | ***p* value** | **NSCLC**  (n=64) | ***p* value** | ***p* value between LAHNSCC and NSCLC** |
| --- | --- | --- | --- | --- | --- | --- | --- |
| **weight** | -1.3 ± 3.9 | **<.001** | -1.6 ± 3.3 | **<.001** | -1.0 ± 4.4 | .08 | .38 |
| **FM** | -0.9 ± 20.5 | .62 | -3.2 ± 24.7 | .32 | 1.2 ± 15.4 | .53 | .23 |
| **FFM** | -0.9 ± 7.1 | .15 | -0.8 ± 7.1 | .36 | -1.0 ± 7.1 | .26 | .90 |
| **HGS** | -4.5 ± 14.3 | **.001** | -6.2 ± 11.2 | **<.001** | -2.9 ± 16.6 | .17 | .19 |

Mean values are presented with ± SD. Bold values denote statistical significance at the p<.05 level. Abbreviations: FM, fat mass; FFM, fat free mass; HGS, handgrip strength.
